# Transcriptome Analysis Suggests the Roles of Long Intergenic Non-coding RNAs in the Growth Performance of Weaned Piglets

**DOI:** 10.3389/fgene.2019.00196

**Published:** 2019-03-18

**Authors:** Lin Chen, Gaoli Shi, Guoting Chen, Jingxuan Li, Mengxun Li, Cheng Zou, Chengchi Fang, Changchun Li

**Affiliations:** ^1^Key Lab of Agricultural Animal Genetics, Breeding and Reproduction of Ministry of Education, College of Animal Sciences and Technology, Huazhong Agricultural University, Wuhan, China; ^2^The Cooperative Innovation Center for Sustainable Pig Production, Wuhan, China

**Keywords:** lincRNA, RNA-seq, muscle and fat development, growth performance, weaned piglets

## Abstract

Long intergenic non-coding RNAs (lincRNAs) have been considered to play a key regulatory role in various biological processes. An increasing number of studies have utilized transcriptome analysis to obtain lincRNAs with functions related to cancer, but lincRNAs affecting growth rates in weaned piglets are rarely described. Although lincRNAs have been systematically identified in various mouse tissues and cell lines, studies of lincRNA in pigs remain rare. Therefore, identifying and characterizing novel lincRNAs affecting the growth performance of weaned piglets is of great importance. Here, we reconstructed 101,988 lincRNA transcripts and identified 1,078 lincRNAs in two groups of longissimus dorsi muscle (LDM) and subcutaneous fat (SF) based on published RNA-seq datasets. These lincRNAs exhibit typical characteristics, such as shorter lengths and lower expression relative to protein-encoding genes. Gene ontology analysis revealed that some lincRNAs could be involved in weaned piglet related processes, such as insulin resistance and the AMPK signaling pathway. We also compared the positional relationship between differentially expressed lincRNAs (DELs) and quantitative trait loci (QTL) and found that some of DELs may play an important role in piglet growth and development. Our work details part of the lincRNAs that may affect the growth performance of weaned piglets and promotes future studies of lincRNAs for molecular-assisted development in weaned piglets.

## Introduction

Over the past decade, given developments in second-generation sequencing technologies, an increasing number of transcriptome studies have revealed a large number of non-coding transcripts in the genome of mammal (Pauli et al., 2012). The expression levels of these genes are somewhat lower than those of protein-coding genes and are often considered to be transcriptional noises (Li et al., 2013). However, as science and technology have advanced, researchers have gradually realized that non-coding transcripts may possess their own unique regulatory mechanisms; these transcripts are called lincRNAs because they are located between genes and have a structure longer than 200 nt (Mizutani et al., 2012). New evidences reveal that, although they have low coding potential, lincRNAs present certain functions in many cellular processes, including gene regulation, apoptosis and embryonic development (Leung et al., 2013). Four functional types of lincRNA, including signal, decoy, guidance and support, have been roughly identified, and most of these transcripts have been studied in mice (Wang and Chang, 2011; Hong et al., 2018). Research involving pig lincRNA is minimal and thus deserves further exploration (Ran et al., 2016).

Weaned piglets, as a very important class of economic animal, are a typical biological model (Fisar and Hroudova, 2016). Newborn weaned piglets have low feed intake, slow growth and high mortality; thus, the piglets cannot gain weight easily (Lv et al., 2018). During the weaning transition, even if the environment and nutrient feed are adjusted, guaranteeing the growth rate of these animals is difficult (Jones et al., 2012). Previous studies have shown that inadequate energy intake and reduced lipid deposition rates are key factors of post-weaning dysplasia in piglets (Snoeck et al., 2004; Jones et al., 2012). We speculate that the development of muscle and fat (the key tissues for piglet development) during the weaning transition may reflect differences in the growth performance of weaned piglets (Yang H. et al., 2016; Albuquerque et al., 2017). In a previous study, pigs showed very obvious obesity, which was not found in other animals (Madeira et al., 2017). The pig model has been used to explore important diseases caused by excess fat, such as obesity, diabetes, coronary heart disease and arteriosclerosis (Robich et al., 2010). Studying the muscle growth of pigs can also enable the breeding of lean pigs to improve pork utilization (Zhang et al., 2015). However, compared with those of human and mouse, research on pig lincRNAs is scarce (De Quattro et al., 2018). The molecular mechanism of the development of muscle and fat in weaned piglets is yet to be explored.

In this study, we first performed a transcriptome assembly containing muscle and fat using data downloaded from GEO accession number GSE65983. We identified a total of 1,078 putative lincRNAs and characterized these lincRNAs. We then analyzed the expression of these lincRNAs in all samples and identified some DELs. We utilized differential expression analysis to explore DELs that may contribute to decreased growth performance in weaned piglets using the RNA-Seq transcriptome, based on the LDM and SF, to analyze the molecular basis of weaned piglet skeletal muscle and fat development. GO analysis was performed on the adjacent protein-coding genes of DELs to predict their function. Real-time quantitative PCR was used to verify the reliability of data analysis. This study not only enriches the lincRNA database for weaned piglet skeletal muscle and fat development, but also provides new insights into the molecular mechanisms affecting the growth performance of weaned piglets.

## Materials and Methods

### Ethics Statement and Data Sources

All the experiments in our study were carried out according to the Key Laboratory of Agricultural Animal Genetics Breeding and Reproduction, Animal Protection and Use Committee, Wuhan 430070. In this study, the piglets used for sampling were all from the same batch and were raised under the same environmental and nutritional conditions (Pilcher et al., 2015). All samples were taken from the same part of subcutaneous back fat between the eighth and last ribs and LDM (Pilcher et al., 2015). 36 RNA-seq datasets from two different kinds of tissues, including LDM and subcutaneous back fat (18 replicates), were obtained from a previously published study and downloaded from the NCBI’s GEO under GEO data set ID GSE65983 (Pilcher et al., 2015). The pig gene annotations were downloaded from ftp://ftp.ensembl.org/pub/release-93/gtf/sus_scrofa. Moreover, the NR reference sequence (RefSeq)NR database was downloaded from ftp://ftp.ncbi.nih.gov/blast/db/.

### RNA-Seq Reads Mapping and Transcriptome Assembly

To ensure that high-quality data is provided to the initial spliced alignment step, the quality of raw reads was evaluated using FastQc (Xiao et al., 2015). Raw reads were filtered and trimmed using Trimmomatic (version0.3.2) with the default parameters (Xiao et al., 2015). The adapter and low-quality reads were removed for the number of mismatches in a read. The remaining high-quality clean reads were then mapped to the pig reference genome (SusScrofa11.1^1^) with the default parameters by the spliced reads aligner HISAT2(version 2.0.1) (Pertea et al., 2016). According to different database construction methods, we selected the corresponding parameters “known-splicesite-infile” and got the sam file containing all mapping readings for each sample. Using samtools software, the sam files were processed and converted into sorted bam files with parameters “view -H” and “sort -o” (Pertea et al., 2016). Finally, to facilitate transcript assembly and quantification, all bam files were assembled into one complete GTF files with the default parameters and merge function of StringTie (version1.2.2) software (Pertea et al., 2016). In addition, by using the assembled GTF file, StringTie software is able to estimate the expression levels of genes and transcripts in all samples for subsequent studies with the parameters “-e” and “-B” (Pertea et al., 2016).

### Pipeline for lincRNA Identification

**Figure 1:**
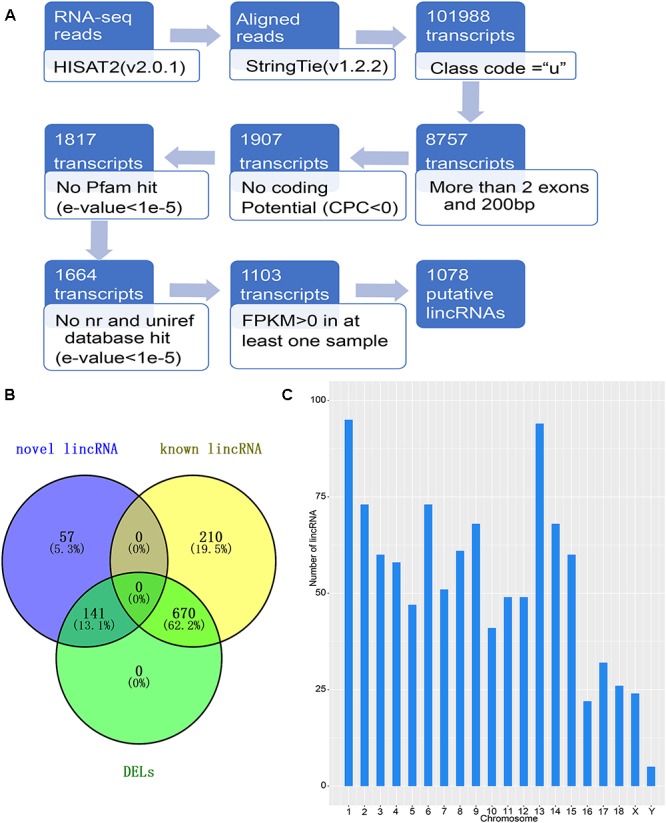
Identification of lincRNA in weaned piglets. **(A)** Overview of the identification pipeline for putative lincRNAs in weaned piglets. **(B)** Venn diagram of all lincRNAs and DELs. **(C)** The number distribution of lincRNA on the chromosome.

The detection of NR transcriptomes for the detection of lincRNAs is basically based on previous laboratory studies (Zou et al., 2017a,b). The principal pipeline employed for lincRNA identification was as follows (Figure 1A): (1) filter transcripts that without ‘u’ characters to retain intergenic transcripts with the parameters “-r” of gffcompare in StringTie (version1.2.2) software (Pertea et al., 2016); (2) remove transcripts with single exons and less than 200 bp in length according to the merged GTF file; (3) collect the positional information of the exons of the remaining transcripts, extract the genomic sequence of the corresponding exon, and fuse into a complete transcript sequence; (4) Use the CPC tool to assess the protein coding potential of complete transcript sequences, retaining transcripts that cannot encode proteins based on protein coding potential (Kong et al., 2007); (5) eliminate transcripts containing any known protein coding domain. Using Transeq^2^ to translate transcript sequences into six possible protein domains and if the corresponding transcripts had a significant hit in the Pfam database^3^, they were discarded by HMMER (*E*-value < 1e-5) (Finn et al., 2015); (6) Using the BLASTX program to filter out any transcript which is similar to a known protein with an *E*-value < 1e-5 in the NCBI, NR, and the UniRef90 database (Pirooznia et al., 2008); (7) Retained transcripts with FPKM values greater than 0 in at least one sample as candidate lincRNAs.

### Comparisons Between lincRNAs and Protein-Coding Transcripts

We selected the protein-encoded character as a marker in the transcript of the Ensemble gene annotation of the pig, resulting in a total of 45,788 protein-coding transcripts. We then identified the protein-coding transcripts and all lincRNA transcripts in terms of length, number and length of exons, and expression levels. Finally, we compared the different characteristics in transcript length, exon length, exon number and FPKM between lincRNA transcripts and protein-encoding gene transcripts and visualized them using R language, with *p*-value less than 0.05 was considered to be significant.

### Analysis of Adjacent Genes

Previous studies have shown that mammalian lncRNAs are preferentially located next to genes with developmental functions (Cabili et al., 2011). Therefore, for each DEL locus the nearest protein-coding neighbor within <100 kb was identified by BEDTools (version 2.17.0), and these neighboring genes are expressed in at least one sample (Quinlan and Hall, 2010). This resulted in a list of lncRNA loci/protein-coding loci pairs. Pearson correlation was used to explore the expression-based relationship between these pairs (Cabili et al., 2011). Ensembl was used to convert the protein-coding gene genes id into human genes id for annotation, visualization and integrated discovery (DAVID) (Huang et al., 2009). Then, we performed GO analysis and KEGG pathway analysis on all expressed protein-coding gene of DELs (Xing et al., 2016). Terms with a *p*-value < 0.05 were considered statistically significant (Benjamini and Cohen, 2017).

### Differentially Expressed lincRNAs Analysis

To perform differential expression analysis on these lincRNAs, we divided 36 samples into two groups, using DEseq2 to generate differential expression of all transcripts (Love et al., 2014). We screened the differentially expressed transcripts by using the fold change with an absolute value greater than 1 and an adjusted *p*-value of less than 0.05, and then crossed the entire lincRNA to obtain DELs. Finally, the results were processed by the DEseq2 package in the R computing environment to produce global gene expression figures and identify differentially expressed protein-coding and lincRNA transcripts based on *p*_adj_ < 0.05 (Benjamini et al., 2001).

### Functional Prediction of DELs by QTL

We further predicted the function of lincRNA by comparing the position of QTL and lincRNA on the whole genome to determine their overlapping regions with BEDTools2.17.0. The QTL database was downloaded from Animal QTL^4^. A total of 26,348 QTLs can be divided into five categories, including (meat and carcass, production, reproduction, health, and exterior) (Zhao et al., 2018).

### Validation of Gene Correlation by qPCR

To verify the association between the protein-coding gene and the lincRNA gene, 10 protein-coding genes and ten lincRNAs were selected for qPCR validation, the corresponding gene sequences were extracted and the gene-specific primers were designed using the Primer5 program. All RNA was extracted from the muscle and fat of three pigs using Trizol reagent (Invitrogen, Life Technologies, Carlsbad, CA, United States) from PSC, and the sample concentration was measured using the Agilent 2100 system. According to the manufacturer’s instructions, SYBR Green (CWBIO, Beijing, China, CW0957) was used as a fluorescent dye, and 18S rRNA was used as an internal reference to detect the lncRNA gene and protein-coding gene in the Roche LightCyler 480 system (Roche, Mannheim, Germany). The delta delta *C*t ([Delta][Delta]Ct) method was used to analyze qPCR data and quantify gene expression (Rao et al., 2013).

## Results

### Genome-Wide Identification of lincRNAs Based on RNA-Seq Datasets

To identify the lincRNAs involved in the muscle and adipose tissue of weaned piglets, we used published data for RNA-seq (Supplementary Table S1) involving two groups of 36 samples (Pilcher et al., 2015). The RNA-seq datasets were sequenced on the Illumina Hiseq2000 platform (Pilcher et al., 2015). And the reads in the datasets are paired, with read lengths exceeding 200 bp (Liu et al., 2018). Based on the pipeline, which is explained in detail in the Section “Materials and Methods,” we obtained new lincRNAs, as shown in Figure 1A. Briefly, beginning from a total of 115.6 million reads, we performed short-read gapped alignment using Hisat2 and recovered 108.5 million mapped reads onto the pig genome version 11.1 (Hong et al., 2018). Then, we used the publicly available assemble software StringTie to *de novo* reconstruct the transcriptome for each tissue based on the read-mapping results (Pauli et al., 2012). The reconstructed transcripts were merged into a single set of transcripts using the merge utility provided by StringTie. We first compared 101,988 transcripts with known genes and obtained a set of 8,757 potential intergenic transcripts. Then, we filtered by the exon number and transcript length and acquired 1,907 potential long intergenic transcripts. Next, we analyzed the coding potential of unannotated transcripts with the CPC tools, which retained 1,817 transcripts. We also scanned each transcript against a six-class protein framework to exclude any transcript that has or may have protein encoding ability. This filtering retained 1,664 potential lincRNA transcripts. We performed a sequence homology search with the BLAST tool and obtained 1,103 potential lincRNA transcripts. Using FPKM expression level of more than 0 at least in one tissue sample as a basis, we finally obtained 1,078 reliably putative lincRNAs, including 198 novel lincRNAs and 880 known lincRNAs (Supplementary Table S3 and Figure 1B). We analyzed the position of each lincRNA and found that most lincRNAs are concentrated on chromosomes 1 and 13, which may be caused by the long length of these two chromosomes (Figure 1C).

### Characteristics of Known and Novel lincRNAs

**Figure 2:**
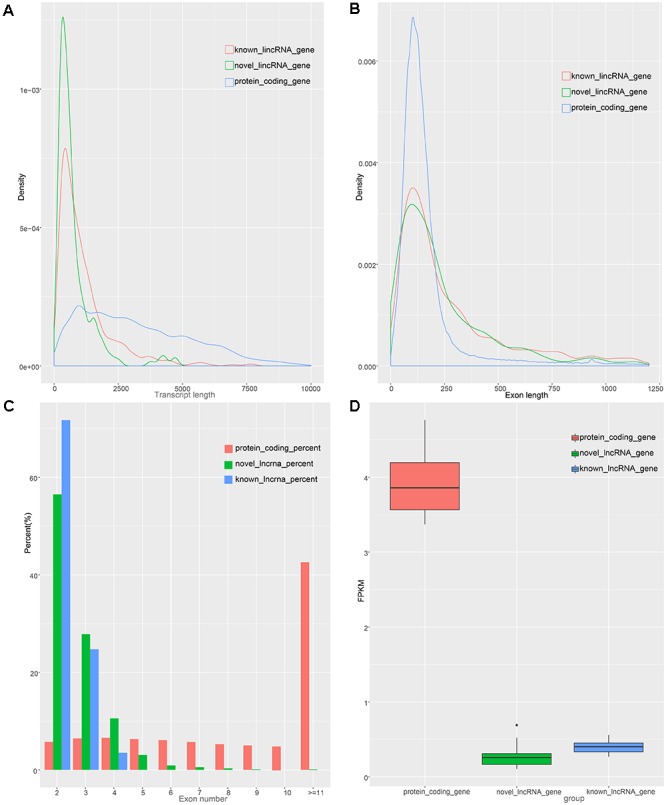
Characteristics of lincRNAs and protein coding gene. **(A)** transcript length distribution of lincRNAs and protein coding gene. **(B)** Distribution of exon length in lincRNAs and protein coding gene. **(C)** Distribution of exon number in lincRNAs and protein coding gene. **(D)** Comparison of expression level between lincRNAs and protein-coding genes.

To identify the characteristics of the lincRNA transcripts, we divided the lincRNA into two major classes, namely known and novel, based on the reconstructed transcriptome and compared them to the obtained protein-coding transcript. According to the Ensembl database, we acquired 45,788 protein-coding transcripts corresponding to the 22,342 protein-coding genes annotated in pigs (Supplementary Table S2), and obtained 12,103 known lincRNA transcripts corresponding to 7,381 lincRNA genes in ALDB. In our study, the average transcript length of the new lincRNA gene was 776 bp, while the average transcript length of the known lincRNA genes was 1,159 bp; these lengths are both shorter than that of protein-coding gene (3,284 bp) (Figure 2A). In term of average exon length, the novel lincRNA gene is 334 bp in length, shorter than the known lincRNA gene (431 bp) but longer than the protein-coding genes (283 bp) (Figure 2B). In addition, we found that the average number of exons in the protein-coding gene is 11.7, which is significantly higher than that of the new lincRNA genes (2.3) and the known lincRNA gene (2.7) (Figure 2C). Because lincRNAs lack protein coding ability, we compared the expression levels of 1,078 lincRNAs with the expression levels of protein-coding genes (Supplementary Table S4). The results showed that the FPKMs of the lincRNA genes and the unknown lincRNA genes are 0.39 and 0.25 (Supplementary Table S5), respectively, which are both lower than the average expression level of the protein-coding gene (FPKM: 3.9) (Supplementary Table S7 and Figure 2D). In general, lincRNA genes exhibit shorter transcription lengths, longer exon lengths, fewer exons, and lower expression levels than protein-encoding genes. All of which, as previously reported, are typical features of lincRNA (Cabili et al., 2011; Djebali et al., 2012).

### Tissue Expression Profile of lincRNAs

**Figure 3:**
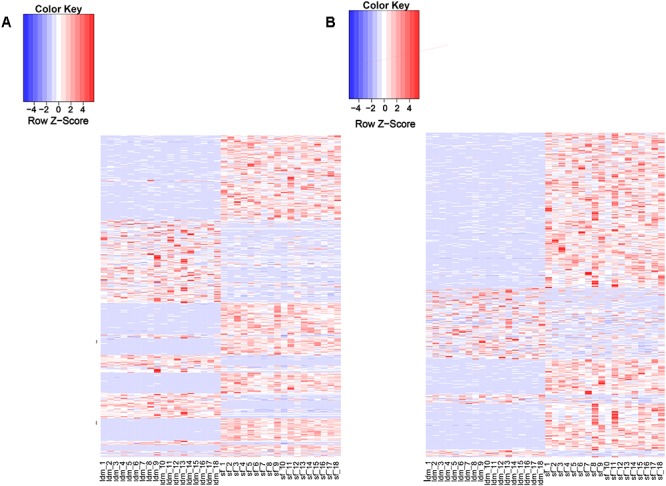
Expression profile of lincRNAs. **(A)** Heat map of differentially expressed genes among two groups. **(B)** Heat map of differentially expressed lincRNAs among two groups. Red, increased expression; white, neutral expression; blue, decreased expression.

Recent studies have shown that lincRNAs are expressed more tissue-specifically than protein-coding genes (Birney et al., 2007; Nakaya et al., 2007). We analyzed the expression patterns of all DELs and protein-encoding genes (Supplementary Table S6). About 97% (1078/1103) of the potential lincRNAs were expressed in more than one sample, and 54.8% (591/1078) lincRNA genes showed differential expression in both tissue types (Supplementary Table S9). By contrast, among all protein-coding genes, only 2.7% (601/22342) were differentially expressed in the two tissues (Supplementary Table S8). The proportion of DEL genes was significantly greater than differentially expressed protein-encoding genes. Therefore, we suggest that the lincRNA genes show a more tissue-specific expression landscape than protein-coding genes (Figure 3A,B). To explore the function of lincRNAs, after profiling 880 known lincRNAs and 198 novel expression in all groups, we conducted the differential expression analysis among the two groups using DEseq2 in the R package and detected 811 DELs. Using *p*-value and fold change as bases, we found 254 up-regulated and 337 down-regulated DELs in the LDM and SF groups.

### Nearest-Neighbor Analysis of lincRNAs

**Figure 4:**
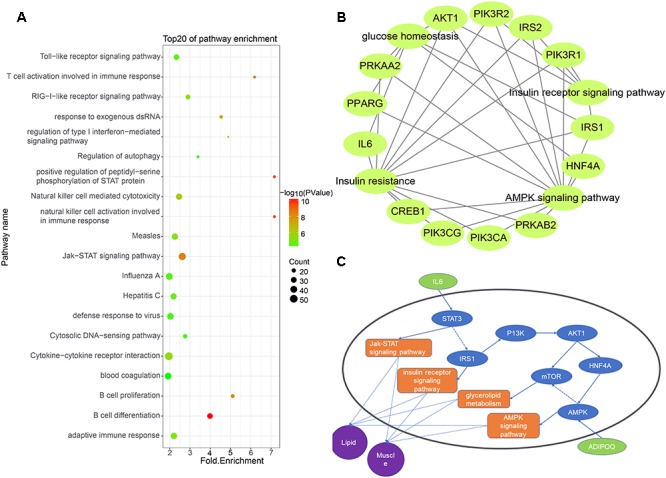
**(A)** Functional enrichment map of potential target genes of differentially expressing lincRNA. **(B)** Genes enriched in muscle and fat pathways. **(C)** Pathway map of potential target genes expressed in muscle and lipid.

Some studies have shown that lincRNAs may regulate their neighboring genes in a *cis*-acting manner (Cabili et al., 2011). To explore the expression relationship between lincRNAs and their neighboring protein-coding genes, we performed GO analysis and KEGG analysis on expressed protein-coding genes transcribed near lincRNA (<100 kb) (Zhao et al., 2015). Using Ensembl and DAVID, we obtained relevant pathway information for these protein-coding genes. The results showed that 955 of 2,251 protein-coding genes significantly participated in 84 biological processes and 37 pathways (Supplementary Table S2). Many protein-coding genes are involved in biological processes or pathways related to muscle growth and fat deposition, such as glucose homeostasis, lipid transport and insulin receptor signaling pathway. Interestingly, at the top 20 of these biological processes and pathways is the Jak-STAT signaling pathway, which corresponds to the biological processes of positive regulation of peptidyl-serine phosphorylation of STAT protein (Figure 4A).

We selected biological processes and pathways related to muscle development and fat deposition from all the results, including glucose homeostasis, glycogen metabolism, lipid transport, cortical actin cytoskeletal organization, the insulin receptor signaling pathways, insulin resistance and the AMPK signaling pathways (Figure 4B,C). Enrichment analysis of the protein-coding genes in these pathways revealed that 12 genes appeared multiple times, with the AKT and IRS1 genes appearing four times in all pathways. According to previous studies, AKT may act on MYOD to affect muscle differentiation (Li X. et al., 2018). Moreover, Li D. et al. (2018) found that lncHR1 can act on AKT to inhibit SREBP1C level and regulate fat metabolism. Zhu et al. (2018) found that miR-146b regulates glucose homeostasis in porcine primary adipocytes by targeting IRS1. These studies indicate that the target genes we found are indeed capable of regulating muscle development and fat metabolism.

### Function Prediction of lincRNA

**Figure 5:**
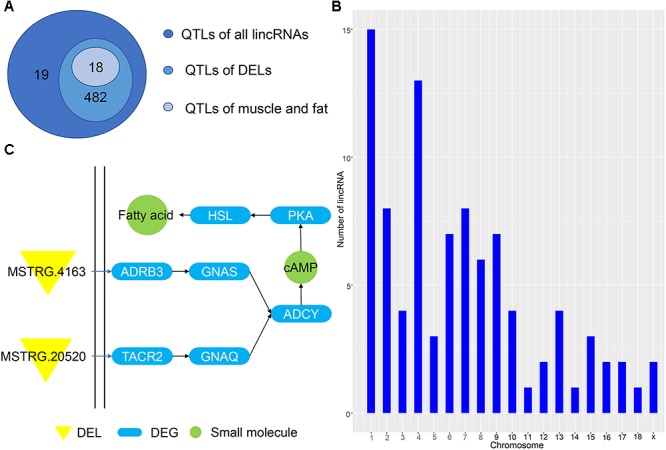
**(A)** QTL of all lincRNAs and DELs. **(B)** Chromosome map of lincRNA for ADG. **(C)** Pathway map of potential target genes of DELs.

Predicting the functions of lincRNAs remains challenging due to their lack of annotation and low expression levels (Han et al., 2016). Here, we analyzed the positional relationship between QTLs and lincRNAs to determine their overlapping regions to infer the function of lincRNA. We compared all lincRNAs and DELs to the porcine QTL database to speculate the function of the identified lincRNA. A total of 1078 lincRNAs overlapped with 501 QTL regions, of which 811 DELs corresponded to 482 QTLs (Figure 5A). Eighteen QTLs had multiple functions, including average daily gain, muscle fiber development and intramuscular fat; all of which are related to the muscle and fat development of weaned piglets. Then, we studied the average daily gain of QTLs and found that 93 lincRNAs fall into the QTL interval and are distributed on all chromosomes (Figure 5B). These lincRNAs are concentrated on chromosomes 1 and 4, consistent with previous results in Figure 1, thereby indicating that most of the identified lincRNAs are associated with average daily gain. In order to understand the specific functions of DEL, we enriched several pathways using GO analysis, the most important of which is the cAMP pathway. In this pathway, we found that the DEL MSTRG.4163 can regulate the differentially expressed potential target genes ADRB3 (Figure 5C). Previous studies have confirmed that activation of ADRB3 stimulates cAMP to act on PKA and promotes lipolysis through HSL phosphorylation (Shin et al., 2016). And cAMP can regulate the expression of AKT to change glucose metabolism (Xie et al., 2014; Arcaro et al., 2018).

### Correlation Validation of lincRNA and mRNA by qPCR

**Figure 6:**
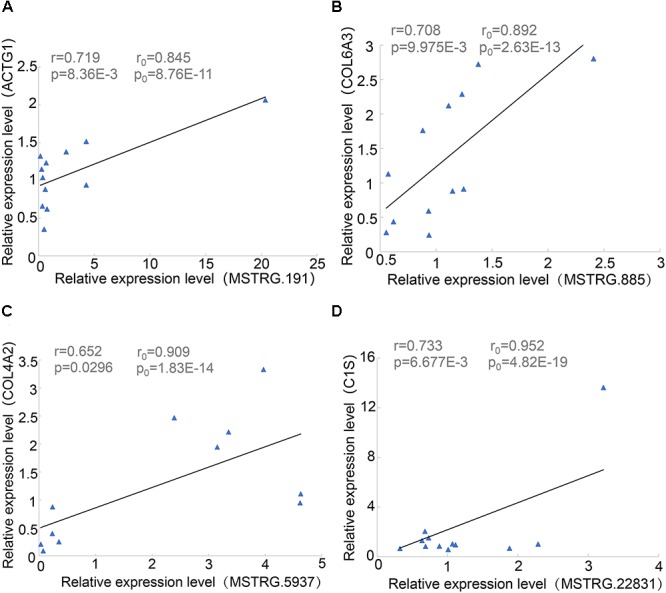
Linear regression of lincRNA and protein-coding gene expression. *R*_0_ and *p*_0_ represent the correlation coefficient and *p*-value of each pair of lincRNA and protein-encoding genes in 18 samples of Pearson; and *r* and *p* represent the verification in 12 samples. **(A)** MSTRG.191 vs. ACTG1. **(B)** MSTRG.885 vs. COL6A3. **(C)** MSTRG.5937 vs. COL4A2. **(D)** MSTRG.22831 vs. ACTG1.

During proximity genetic analysis, we predicted potential target genes that differentially express lincRNAs. To verify the reliability of the data analysis, we selected four pairs of lincRNA genes and protein-coding genes for real-time quantitative PCR. The lincRNAs were differentially expressed, while COL6A3, COL4A2, and C1S were highly expressed in SF, and four protein-coding genes were expressed in LDM. The experimental results showed that the *p*-values of the four pairs of genes were less than 0.05, the Pearson correlation coefficient of COL4A2 and MSTRG.5937 was 0.65, and the Pearson correlation coefficient of C1S and MSTRG.22831 was 0.733 (Figure 6). The correlation coefficient and *p*-values of the genes were calculated according to their expression level in the original data set, and their relationship was basically consistent with the basic trend of experimental verification.

## Discussion

In this study, we performed a comprehensive analysis of 36 RNA-seq libraries based on the designed pipeline and we identified 1078 lincRNAs in LDM and SF of weaned piglets. Compared with the known lincRNA database, we found 198 new putative lincRNAs. To detect the genomic characteristics of the identified lncRNA, we compared the known and novel lincRNAs with the porcine protein-coding gene and found that lincRNAs have shorter transcripts, fewer exons, longer exon lengths and lower expression levels (Koufariotis et al., 2015; Han et al., 2016; Yang M. et al., 2016). Previous studies have shown that the size of the pig genome is essentially the same as that of humans and mice; however, fewer lincRNAs have been identified in pigs than in the above two species, thereby indicating that a large number of lincRNAs in pigs have not been found (Kwok and Tay, 2017; Zhou et al., 2017). Therefore, the 198 new putative lincRNAs identified in this work not only enrich the lincRNA database but also extend the lincRNA annotation information of pigs.

Muscle and adipose, the two most important tissues in animals, are closely related to the growth and development of organisms (Kociucka et al., 2017; Liu et al., 2017). Muscle and fat development can affect the growth rate of livestock, meat production and meat quality, and also cause diseases such as obesity in human. Studying the developmental mechanisms of muscle and fat is important to obtain an in-depth understanding of the above issues. To study the relationship between the development of two tissues and the growth performance of piglets, we focused on differential expression analysis. By comparing DELs in weaned piglets between the two tissue types, we can effectively identify important lincRNA genes during the growth process. Analysis of the expression of these lincRNAs in muscle and fat promotes the understanding of their tissue specificity and potential function. We identified a total of 811 DELs, corresponding to 591 lincRNA genes between muscle and adipose tissue that may result in differences in growth performance. To predict the function of these lincRNAs, we examined their potential target genes in the vicinity of the 100 k region of DELs. However, the mechanism by which lincRNA regulates its potential target genes remains unclear and requires further investigation.

Enrichment analysis revealed that the processes and pathways enriched by potential target genes directly or indirectly affects muscle and fat development (Ayuso et al., 2015; Ibanez-Escriche et al., 2016). Several reports that lincRNA may have *cis*-regulatory relationships with these potential protein-coding genes have been published (Quattro et al., 2017). Some lincRNAs have be speculated to play an regulatory role in muscle development and fat deposition, which can change the expression pattern of target genes and promote the growth of weaned piglets. Three pathways are related to muscle and fat, namely glucose homeostasis, the AMPK signaling pathway and the insulin receptor signaling pathway. Previous studies have reported that glucose homeostasis can maintain the normal metabolism of cells, ensure the proliferation and differentiation of muscle cells and promote the formation of muscle fibers (Lauritsen et al., 2002). As a cellular energy receptor, AMPK responds to low levels of ATP and, when activated, positively regulates the signal transduction pathway that complements cellular ATP supply (Scheffler and Gerrard, 2016). The insulin receptor signaling pathway, once blocked, weakens the function of islet B cells and forms a large number of free fatty acids, leading to the dysregulation of fat and sugar metabolism in the body. Over time, the body forms fat deposits (Rajala and Anderson, 2010). These pathways and biological processes can affect the formation of muscle and adipose tissue to some extent. Although the mechanisms by which these lincRNAs regulate their potential target genes are unclear, these enriched biological processes suggest that some of the lincRNAs we identified may have functions that regulate muscle growth and fat metabolism. Our study provides new insights into the discovery and annotation of lincRNAs associated with growth performance in weaned piglets, thereby providing an opportunity to further explore the molecular mechanisms underlying pig growth and development.

In this study, we also performed a simple QTL analysis to locate lincRNAs on QTLs. Since most QTLs are regulated by multiple genes, although studies have identified major genes by mapping QTL maps, accurate identification of target genes corresponding to a certain lincRNA remains challenging (Grisart et al., 2002). Moreover, most QTLs are fairly large, whereas lincRNAs are relatively short. A large number of lincRNAs may fall on the same QTL, causing difficulties in predicting the functions of specific lincRNAs. Based on the regulation of lincRNAs to their neighboring genes (<100 k), we may be able to predict the functions of lincRNAs more accurately. In this paper, we studied the average daily gain of QTLs and found that 93 lincRNAs fall into the QTL interval and are distributed on all chromosomes, but only 2 lincRNAs correspond to QTL intervals less than 100 kb. MSTRG.9938.1 is located on chromosome 15, almost coinciding with the QTL interval of 40 b, and is more likely to affect the average daily gain of piglets than MSTRG.4760.27, which corresponds to the 66 kb QTL interval on chromosome 12. Although our QTL study cannot directly determine the functions of lincRNAs, it can screen out a number of lincRNAs that may be associated with different production traits. This result helps to enrich the genetic database associated with piglet growth performance and lays the foundation for improved piglet performance.

In summary, we identified and characterized several lincRNAs involved in muscle and fat development in weaned piglets. Tissue-specific analysis identified partially DELs, and adjacent gene analysis and functional enrichment revealed that many lincRNAs may act on potential target genes to participate in muscle growth and lipid deposition-related processes. QTL positional alignment also indicated that these genes can act on muscle or fat to further regulate average daily gains. Given that the functions and molecular mechanisms of lincRNAs are unclear and that lincRNAs annotated on pigs are not complete, this work provides a valuable resource for enriching the pig lincRNA database and helps improve the understanding on the potential functions of lincRNAs during the weaning transition phase for muscle and fat development.

## Data Availability

Publicly available datasets were analyzed in this study. This data can be found here: https://www.ncbi.nlm.nih.gov/Traces/study/?acc=PRJNA275632.

## Author Contributions

CL conceived and designed the experiments and explained the data. LC analyzed the main content of the data with the help of GS, GC, CZ, and CF. JL performed the experiments with the help of ML. LC wrote the manuscript with the help of CL.

## Conflict of Interest Statement

The authors declare that the research was conducted in the absence of any commercial or financial relationships that could be construed as a potential conflict of interest.

## Footnotes

1 ftp://ftp.ensembl.org/pub/release-93/fasta/sus_scrofa/dna/

2 https://www.ebi.ac.uk/Tools/st/emboss_transeq/

3 http://pfam.xfam.org/search

4 https://www.animalgenome.org/cgi-bin/QTLdb/SS/index

## Supplementary Material

TABLE S1 Summary of data from RNA-seq.

TABLE S2 GTF of protein coding gene.

TABLE S3 GTF of lincRNA gene.

TABLE S4 FPKM of all transcripts.

TABLE S5 FPKM of all lincRNA transcripts.

TABLE S6 Count value of all genes.

TABLE S7 FPKM of protein coding gene.

TABLE S8 Count value of all differentially expressed genes.

TABLE S9 Count value of all differentially expressed lincRNA genes.

## Abbreviations

ALDB: the domestic-animal lncRNA database
CPC: coded potential calculator
DAVID: database for annotation, visualization and integrated discovery
DELs: differentially expressed lincRNAs
FPKM: fragments per kilobase of transcript per million mapped reads
GEO: gene expression omnibus
GO: Gene Ontology
GTF: gene transfer format
KEGG: Kyoto Encyclopedia of Genes and Genomes
LDM: longissimus dorsi muscle
lincRNAs: long intergenic non-coding RNAs
NCBI: National Center for Biotechnology Information
NR: non-redundant
QTL: Quantitative Trait Locus
RT-qPCR: real-time quantitative polymerase chain reaction
SF: subcutaneous fat
